# Weekly Physical Activity Levels of Older Adults Regularly Using a Fitness Facility

**DOI:** 10.1155/2016/5010285

**Published:** 2016-05-18

**Authors:** Michael J. Turner, Emily E. Schmitt, Tricia Hubbard-Turner

**Affiliations:** ^1^Laboratory of Systems Physiology, University of North Carolina at Charlotte, Charlotte, NC 28223, USA; ^2^Biology of Physical Activity Laboratory, Texas A&M University, College Station, TX 77843, USA; ^3^Biodynamics Research Laboratory, Department of Kinesiology, University of North Carolina at Charlotte, Charlotte, NC 28223, USA; ^4^Center for Biomedical Engineering & Science, University of North Carolina at Charlotte, Charlotte, NC 28223, USA

## Abstract

The aim of this paper was to determine if weekly physical activity levels were greater in an independent-living older adult population that was regularly participating in structured fitness activities. Also, lifetime exercise history and sex differences were investigated in an effort to understand how they relate to current weekly step activity. Total weekly step counts, measured with a pedometer, were assessed in two older adult groups; the first consisted of members of a local senior center who regularly used the fitness facility (74.5 ± 6.0 yrs; mean ± SD) while the second group consisted of members who did not use the fitness facility (74.8 ± 6.0 yrs). Participants also completed the Lifetime Physical Activity Questionnaire (LPAQ). No significant difference was found in the total number of weekly steps between groups (*p* = 0.88) or sexes (*p* = 0.27). The LPAQ suggested a significant decline in activity with aging (*p* = 0.01) but no difference between groups (*p* = 0.54) or sexes (*p* = 0.80). A relationship was observed between current step activity and MET expenditure over the past year (*p* = 0.008, *r* ^2^ = 0.153) and from ages 35 to 50 years (*p* = 0.037, *r* ^2^ = 0.097). The lack of difference in weekly physical activity level between our groups suggests that independent-living older adults will seek out and perform their desired activity, in either a scheduled exercise program or other leisure-time activities. Also, the best predictor of current physical activity level in independent-living older adults was the activity performed over the past year.

## 1. Introduction

Maintaining aerobic capacity and increasing muscular strength are known to lead to improvements in overall function in the aging population. An increase or maintenance of regular physical activity levels with aging would likely aid in the improvement in the functional capacity of the older adult. After 16 weeks of an exercise program focusing on improving aerobic capacity, muscular strength, and muscular endurance, Fahlman et al. [1] reported improvements in older adults on measures of aerobic fitness and functional ability. Nakamura et al. [2] demonstrated similar results in which participation in physical activity (walking, recreational activities, and resistance training) at least three times a week over a twelve-week period led to improvements in body composition. Importantly, participation in a regular structured exercise program has been found to improve health and well-being [1–3] and lead to an increase in the amount of physical activity performed outside of the exercise class on nonstructured exercise days [4]. Therefore, participation in regular structured exercise programming would likely benefit the aging population.

In addition, past research has shown that those who participate in exercise during youth and adolescent years have increased exercise levels in adulthood and lower body mass index levels [5, 6]. Those who report moderate to vigorous physical activity in midlife have been observed to possess better mobility later in life [6]. However, research is limited in this area; therefore identifying possible relationships between past exercise and current fitness levels remains an important goal for gerontological studies. In the current study a sample of both males and females were studied, ranging in age from 65 to 95 years. The use of a Lifetime Physical Activity Questionnaire [7] allowed for a look at physical activity across the lifespan to provide evidence as to whether past exercise can be used as a predictor of future levels of physical activity in later adulthood.

The purpose of this study was to assess total weekly steps taken in two older adult groups who frequent a local senior center using a pedometer. The first group consisted of members of a senior center who used the fitness facility at least two times a week (FIT). The second group consisted of members who have never used a fitness facility (NFIT). We hypothesized that those who participate in using the exercise facility at a senior center would have a greater total number of steps than those who did not use the fitness facility. Also, we hypothesized that those who regularly participated in exercise earlier in life would perform a greater number of total steps compared to those less active earlier in life. Finally, based on the past literature [6, 8–11] we hypothesized that males would exhibit a greater number of weekly steps compared to the female participants.

## 2. Methods

### 2.1. Participants

Eighteen males and 27 females ranging in age from 65 to 95 years were recruited from the Charlotte-Mecklenburg Senior Center in Charlotte, North Carolina. This study consisted of mostly European American participants (*n* = 42), two African Americans (*n* = 2), and one Asian (*n* = 1). Volunteers were recruited from advertising for interested individuals who regularly frequented the facility. This study was approved by the Institutional Review Board of the University of North Carolina at Charlotte.

### 2.2. Study Design

Participants were placed into one of two groups. The first group consisted of 13 males and 16 females who were members of the senior center and regularly used the fitness facility at least two times a week (FIT). The second group was composed of 5 males and 11 females who were members of the senior center but never used a fitness center, either in the senior center or elsewhere (NFIT).

The potential benefits and risks of participating in this study were fully explained to each participant. Participants were required to complete a medical history form and provide written informed consent before receiving a pedometer to measure daily steps for one full week. Subjects were also instructed about completing the Lifetime Physical Activity Questionnaire (LPAQ) [7] throughout the one-week period during which daily steps were measured. Height (cm) and body mass (kg) were recorded at the start of the study period, with body mass also recorded on the last day of the study period to monitor any changes. Subject's body mass (kg) was measured on a standard scale (J.A. King and Company, Inc., Greensboro, NC). From the height and initial mass measurements, body mass index (BMI) was calculated in kg/m^2^.

### 2.3. Measurement of Physical Activity Levels

#### 2.3.1. Pedometers

All volunteers were provided with a Digi-Walker SW-200 pedometer (New Lifestyles, Inc., Lees Summit, Missouri) at the beginning of the study period. Subjects were instructed on the proper way to wear the pedometer (on the waistband or belt close to the hip joint) and were reminded to wear the device at all times except when bathing, showering, swimming, or sleeping at night. Participants were asked to put on the pedometer first thing in the morning and remove the pedometer from the belt or waistline, complete the provided daily step log, and zero the pedometer before going to bed for seven consecutive days. Daily step totals were added together and the calculated sum equaled the weekly total for each participant. All subjects were asked to maintain their typical daily routine and leisure activities for the duration of the study.

#### 2.3.2. Lifetime Physical Activity Questionnaire (LPAQ)

Each subject was given a LPAQ [7] on the first day of the study. They were instructed on how to complete the questionnaire and were asked to fill it out and return it at the end of the study period with the pedometer. When the participant returned the LPAQ [7], the completed questionnaire was reviewed with the principal investigator. Intensity was determined by asking each participant how intensely they were working while performing a certain activity. Intensity was measured in multiples of metabolic equivalents (MET, 3.5 mL/kg/min) with intensity levels being assigned using the* Compendium of Physical Activities* [12].

The data from the LPAQ [7] were scored by calculating the minutes per year a person was performing a certain activity multiplied by the MET levels assigned to that activity. Total MET expenditure was determined for each age period (15–21 years, 22–34 years, 35–50 years, 51–65 years, and the past year) and for total lifetime.

### 2.4. Statistical Analyses

All descriptive data were analyzed using a one-way analysis of variance between groups. A two-way analysis of variance by group and sex was used to determine any differences in the total number of weekly steps taken between groups (FIT versus NFIT) and between males and females. Group changes in body mass during the study period were compared using a two-way analysis of variance with repeated measures. Any relationships between the LPAQ [7] MET levels for each age period and current weekly step count were determined by performing linear squares regression analyses for all participants and for each group. Also, linear regression analyses were performed for all participants and for each group between pre body mass and BMI versus total weekly steps. The LPAQ [7] MET levels for each age period were compared by two-way analysis of variance with repeated measures between groups (group × age) and sexes (sex × age). All statistical conclusions were based on an alpha level of 0.05 and performed using JMP Statistical Analysis software (SAS Institute, Cary, NC).

## 3. Results

### 3.1. Subject Characteristics

A total of 29 people in the FIT group (female *n* = 16, male *n* = 13) and a total of 16 people in the NFIT group (female *n* = 11, male *n* = 5) participated in the study. There was no difference in age between the two groups (*p* = 0.88). A summary of the descriptive data can be found in Table 1. Also, there was no difference in height between groups (*p* = 0.22). There was no difference between groups for pre body mass (*p* = 0.58), post body mass (*p* = 0.60), or across time (*p* = 0.55). However, BMI was found to be different between groups (*p* = 0.05), with FIT exhibiting a greater BMI than the NFIT group.

### 3.2. Total Number of Weekly Steps

**Figure 1:**
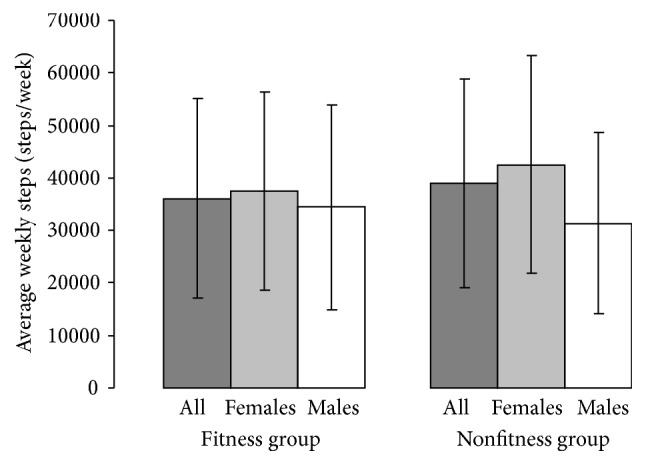
Mean (±SD) of average weekly steps for the participants in the fitness and nonfitness group.

Analysis of total number of weekly steps between FIT and NFIT exhibited no significant difference (*p* = 0.88; Figure 1). No difference in total number of weekly steps was observed between males and females (*p* = 0.27). Also, no interaction was observed for total weekly steps for sex and group (*p* = 0.53).

### 3.3. Lifetime Physical Activity Questionnaire MET Levels

A two-way analysis of variance for group and age with Lifetime Physical Activity Questionnaire (LPAQ) [7] MET levels found no difference between groups (*p* = 0.54) but a significant decrease in activity level with age (*p* = 0.01). There was no significant interaction effect (*p* = 0.37).

A two-way analysis of variance for sex and age with the LPAQ [7] found no difference between sexes (*p* = 0.80), a significant decrease with age (*p* = 0.01), and a significant interaction (*p* = 0.003). The sex by age interaction effect suggests that male activity decline begins after 22–34 years while female activity declines after 35–50 years.

### 3.4. Comparing Total Weekly Steps and LPAQ MET Levels

Linear least squares regression was performed between total weekly steps and the different age periods with the LPAQ METs per year [7]. Total weekly steps were significantly related for all subjects to the past year MET levels (*p* = 0.008; *r* ^2^ = 0.153) and 35–50 yr age period (*p* = 0.037; *r* ^2^ = 0.097). There were no other relationships between total weekly steps and the remaining age periods. All *r* ^2^ values are displayed in Table 2.

For FIT participants, total weekly steps were significantly related with past year MET expenditure (*p* = 0.038; *r* ^2^ = 0.150). For NFIT participants, total weekly steps were significantly related to past year MET expenditures (*p* = 0.047; *r* ^2^ = 0.254). There were no other relationships between total weekly steps and the remaining age periods within each group. All *r* ^2^ values are displayed in Table 2.

### 3.5. Comparing Pre Body Mass and BMI with Total Weekly Steps

Linear least squares regression values between pre body mass and total weekly steps for all participants were not significantly related (*p* = 0.78; *r* ^2^ = 0.002). For the FIT group, pre body mass and total weekly steps were not significantly correlated (*p* = 0.93; *r* ^2^ = 0.0003). With all NFIT participants pre body mass was not related with total weekly steps (*p* = 0.39; *r* ^2^ = 0.053).

There was no significant relationship between BMI and total weekly steps for all participants (*p* = 0.63; *r* ^2^ = 0.006). For FIT, BMI was not related with total weekly steps (*p* = 0.95; *r* ^2^ = 0.0002). Additionally, BMI was not related to total weekly steps for the NFIT group (*p* = 0.38; *r* ^2^ = 0.055).

## 4. Discussion

We found no difference in total number of weekly steps between older adults who regularly participate in the exercise facility at a senior center and those who do not use an exercise facility. Depending on the age period, the current study found a relationship between current steps per week and past activity levels over the past year and from the age period of 35–50 years. Additionally, both groups (i.e., FIT and NFIT) were analyzed by sex to determine any sex-related differences in regular physical activity level. No sex-related differences in physical activity level were observed in this study.

Our first hypothesis was not supported by the findings, which indicate no significant difference in the total number of weekly steps taken between those who regularly use and those who do not use an exercise facility (Figure 1). To our knowledge this is the first study to compare weekly step counts in two groups of older adults who do and do not participate in structured exercise. The findings of the current study may be a result of the observations of Whaley and Ebbeck [13] who interviewed older adults at a senior center to find perceived constraints to participating in structured exercise. Nine females and eight males were interviewed to better understand perceived barriers to exercise class participation. The authors reported that older adults might choose to not participate in structured exercise because they are frequently active elsewhere. The researchers found that some older adults would rather walk around the neighborhood, swim, or ride bikes [13]. They also discovered that older adults claimed that they were too busy to exercise or exercise conflicted with other appointments. Some found structured exercise to be inconvenient [13].

Several studies have outlined how important it is for older adults to participate in structured exercise in order to meet exercise guidelines. Rejeski et al. [14] examined the influence of a physical activity intervention program on satisfaction with self-efficacy along with physical function for older adults who ranged in age from 70 to 89 years. The researchers found that older adults who participated in physical activity (aerobic, strength, balance, and flexibility) had better profiles for satisfaction with physical function and self-efficacy for the 400-meter walk compared to those in a successful aging program. In addition, Tudor-Locke et al. [4] found that older adults who participated in structured exercise experienced an increase in physical activity on the exercise program days relative to other days of the week. The researchers found that walking for exercise was the most prevalent form of exercise outside of a structured exercise class [4].

A previous study assessing physical activity levels of older adults using a pedometer recruited 415 participants [15]. Strath et al. [15] wanted to examine walking volume in older adults by examining personal characteristics associated with walking behavior. These authors showed that race and ethnicity significantly influenced one's average steps per day. They noted that African American older adults took approximately 750 fewer steps per day than European American of the same age [15]. The demographics of our FIT and NFIT groups did not differ significantly in this study and consisted of 42 European Americans, two African Americans, and one Asian participant(s). The current study did not recruit a wide range of ethnic diversity, which could be why there was no statistically significant difference between races.

The current study also found that body mass and BMI did not relate to weekly step count in an older adult population. Body mass and total weekly step counts were not significantly related for all study participants (*p* = 0.78). Scott et al. [16] investigated the effects of pedometer use as a means of encouraging and assessing ambulatory activity in a group of older adults. This study was part of a larger cohort study (Tasmanian Older Adult Cohort Study) to look at the progression of osteoarthritis and osteoporosis in older adults aged 50 to 79 years. These authors [16] observed a negative relationship between body fatness and daily step activity (*p* < 0.001, *r* = −0.54). Although we observed no relationship of step activity and BMI for any of the groups, past studies [16, 17] suggesting body fatness or BMI influencing step activity recruited significantly larger and more heterogeneous sample sizes compared to the current study. These studies incorporated younger adults and individuals with a larger variance in body composition compared to the current study population. Therefore, lack of a relationship in weekly step counts and BMI could be attributed to the lack of a large number of overweight or obese subjects in our groups. Strath et al. [15] reported that older adults considered overweight took about 2,000 steps less than those in the normal weight category while those in the obese category took about 2,500 fewer steps than those in the normal weight category. With only 11.1% of the males and 6.6% of the females in the current study being classified as obese with respect to their BMI, we expect that the high number of normal BMI subjects could be influencing our lack of relationship between BMI and total weekly steps.

The second hypothesis of a relationship between past activity levels and current steps per week was only supported by past year MET expenditure and MET expenditure from ages 35 to 50 years. Other studies have found that past sport participation in childhood can lead to lower BMI values in adult women [5]. Also, results from Patel et al. [6] who studied physical activity levels during three age periods in life (20–40 years, 40–60 years, and the past year) indicated that physical activity performed during two of the age periods (ages 20–40 and 40–60 years) was associated with better mobility and slower onset of chronic disease states in older adulthood determined by functional assessments.

The current study observed a significant decrease in activity level with an increase in age. This is in line with past studies [6, 8, 18, 19]. Norman et al. [19] conducted a study that examined recalled physical activity at 15, 30, and 50 years of age in a large sample of older Swedish men. It was concluded that a decrease in physical activity over time was due to a reduction in leisure-time activities [19]. Patel et al. [6] reported that physical activity levels in older adults over the past year were significantly lower than levels reported for midlife. The Centers for Disease Control and Prevention (CDC) has reported the prevalence of leisure-time physical activity declined for the US population from the years 1994 to 2004 [18]. The largest decline was in the older adult population (men aged 50–59 years and women aged 60–69 years) [18]. In addition, Armstrong et al. [8] showed that men and women generally had declining levels of activity across the lifespan.

Friedman et al. [20] argue that activity levels are somewhat stable from childhood into middle and late adulthood. Friedman et al. [20] continued the Terman Life-Cycle Study, which was started in 1922 by Lewis Terman. The original Life-Cycle Study was composed of 1528 mostly middle class European American boys and girls. Subjects' activity levels were followed throughout their lives with assessments every 5 to 10 years. Data were reviewed for subjects born between the years 1904–1915. The researchers concluded that, after studying significant associations across almost six decades, active children grow into active, energetic adults. Friedman et al. [20] also argued that it is not important to know whether individuals are more active than others, but it is more beneficial to understand past activity levels from the last decade in order to understand current activity levels. Our findings are in agreement with this statement by Friedman et al. [20] with the relationship of current activity levels matching past year's MET expenditures.

Lastly, the current study observed no sex differences in total weekly steps between males and females whether they were in the FIT or NFIT group. This finding does not agree with most research. Past research generally indicates that men are more active than women [6, 8–11]. Lee [10] found that women were significantly less active than men in duration of exercise. The study also noted that 26% of older adult males and 12% of older adult females engaged in the recommended amount of physical activity (30 minutes a day, 5 days a week) [10]. In addition, Yasunaga et al. [11] measured physical activity levels in older adults and concluded that older adult men accumulated more step counts than their female counterparts. Armstrong et al. [8] found that walking was greater in older adult males compared to older adult females. However, recent reviews of physical activity by Friedman et al. [20] and Strath et al. [15] reported little difference between sexes in older adult populations. Friedman et al. [20] found patterns of physical activity across the lifespan to be about the same for males and females. Strath et al. [15] found no significant differences between males and females in any race or ethnic category when investigating steps per day in an older adult population.

### 4.1. Study Limitations

Limitations of this study include the sample population. This research was conducted at a senior center where the majority of individuals were very independent and mostly of high socioeconomic status. Also, the population was all healthy individuals who did not exhibit a wide range of BMI values, with only 11.1% of males and 6.6% of females being categorized as obese. The CDC national averages for BMI in the older adult population show that 32.2% of men and 30.5% of women over the age of 65 years are obese [19]. Additionally, there were few males in the NFIT group. The pedometer was used for the daily assessment of physical activity in our older adult population. Recently Colbert et al. [21] compared the validity values of physical activity measures in older adults using three different activity models. The authors discovered that more expensive devices did not rank physical activity any better than the pedometer, and the pedometer was the most cost-effective method for ranking physical activity level in older adults [21].

Another limitation of this study was the Lifetime Physical Activity Questionnaire (LPAQ) [7]. This questionnaire was used to assess physical and leisure-time activities throughout the lifespan. The decades in which the questionnaire asked about were past activity amounts categorized into different age periods (15–21 years, 22–34 years, 35–50 years, 51–65 years, and the past year). Certain activities and ages may have been missed with this survey tool. For example, an individual who filled out this survey who was 72 years of age was not asked about the age period of 66–71 years. This creates age gaps that could miss pertinent information in regard to lifetime physical activity. Also, with any questionnaire recall can be a problem. Asking an older adult to recall activities they did decades earlier can certainly be a difficult task, especially with regard to intensity levels.

## 5. Conclusions

In conclusion, no difference was observed in the total number of weekly steps taken by older adults who regularly participate at an exercise facility and those who do not use an exercise facility. This finding suggests that independent-living older adults will seek out and perform weekly physical activity, whether in a scheduled exercise program or with other leisure-time activities. To our knowledge the current study is the first to compare weekly step counts in two groups of older adults who do and do not participate in structured exercise. In addition, using questionnaire recall with the LPAQ [7] we observed that physical activity declined with age (3, 5, 15, and 17) [6, 8, 19]. Using the LPAQ [7] we only observed a relationship between current steps per week and MET expenditure over the past year and from ages 35 to 50 years. Also, the current study observed no sex differences in weekly step count between males and females, for all participants or in the FIT or NFIT group.

## Figures and Tables

**Table 1:** Mean (±SD) descriptive characteristics of fitness and nonfitness participants.

	Fitness	Nonfitness
Age (yr)		
All	74.5 ± 6.0	74.8 ± 6.0
Female	73.8 ± 6.2	73.6 ± 5.4
Male	75.4 ± 5.9	77.4 ± 7.3
Height (cm)		
All	168.7 ± 9.7	165.4 ± 8.1
Female	161.5 ± 5.6	161.5 ± 5.6
Male	177.5 ± 4.6	173.2 ± 7.1
Pre mass (kg)		
All	73.3 ± 14.3	75.5 ± 9.4
Female	63.6 ± 7.1	74.5 ± 10.3
Male	85.1 ± 11.6	77.7 ± 7.7
Post mass (kg)		
All	73.5 ± 14.4	75.4 ± 9.4
Female	63.7 ± 7.1	74.2 ± 10.2
Male	85.5 ± 11.6	77.9 ± 7.6
BMI (kg/m^2^)^*∗*^		
All	25.6 ± 3.3	27.7 ± 3.9
Female	24.3 ± 2.3	28.5 ± 3.8
Male	27.0 ± 3.8	26.1 ± 3.9

^*∗*^Denoting significant difference between groups (*p* = 0.05).

**Table 2:** Correlation table (*r* ^2^ values) of total weekly steps and LPAQ MET levels for all subjects and each group.

	Past year	51–65 yrs	35–50 yrs	22–34 yrs	15–21 yrs
All	0.153^*∗*^	0.040	0.097^*∗*^	0.034	0.010
Fitness	0.150^*∗*^	0.030	0.098	0.017	0.037
Nonfitness	0.254^*∗*^	0.057	0.095	0.132	0.030

^*∗*^Denoting significant relationship with total weekly steps (*p* < 0.05).

## References

[1] Fahlman M. M., Topp R., McNevin N., Morgan A. L., Boardley D. J. (2007). Structured exercise in older adults with limited functional ability. *Journal of Gerontological Nursing*.

[2] Nakamura Y., Tanaka K., Yabushita N., Sakai T., Shigematsu R. (2007). Effects of exercise frequency on functional fitness in older adult women. *Archives of Gerontology and Geriatrics*.

[3] Fox K. R., Stathi A., McKenna J., Davis M. G. (2007). Physical activity and mental well-being in older people participating in the Better Ageing Project. *European Journal of Applied Physiology*.

[4] Tudor-Locke C., Jones G. R., Myers A. M., Paterson D. H., Ecclestone N. A. (2002). Contribution of structured exercise class participation and informal walking for exercise to daily physical activity in community-dwelling older adults. *Research Quarterly for Exercise and Sport*.

[5] Alfano C. M., Klesges R. C., Murray D. M., Beech B. M., McClanahan B. S. (2002). History of sport participation in relation to obesity and related health behaviors in women. *Preventive Medicine*.

[6] Patel K. V., Coppin A. K., Manini T. M. (2006). Midlife Physical Activity and Mobility in Older Age. The InCHIANTI Study. *American Journal of Preventive Medicine*.

[7] Chasan-Taber L., Erickson J. B., McBride J. W., Nasca P. C., Chasan-Taber S., Freedson P. S. (2002). Reproducibility of a self-administered lifetime physical activity questionnaire among female college alumnae. *American Journal of Epidemiology*.

[8] Armstrong T., Bauman A., Davies J. (2000). *Physical Activity Patterns of Australian Adults: Results of the 1999 National Physical Activity Survey*.

[9] Kavanagh A. M., Bentley R. (2008). Walking: a gender issue?. *Australian Journal of Social Issues*.

[10] Lee Y.-S. (2005). Gender differences in physical activity and walking among older adults. *Journal of Women and Aging*.

[11] Yasunaga A., Togo F., Watanabe E. (2008). Sex, age, season, and habitual physical activity of older Japanese: the Nakanojo Study. *Journal of Aging and Physical Activity*.

[12] Ainsworth B. E., Haskell W. L., Whitt M. C. (2000). Compendium of physical activities: an update of activity codes and MET intensities. *Medicine and Science in Sports and Exercise*.

[13] Whaley D. E., Ebbeck V. (1997). Older adults' constraints to participation in structured: exercise classes. *Journal of Aging and Physical Activity*.

[14] Rejeski W. J., King A. C., Katula J. A. (2008). Physical activity in prefrail older adults: confidence and satisfaction related to physical function. *Journals of Gerontology: Psychological Sciences and Social Sciences*.

[15] Strath S. J., Swartz A. M., Cashin S. E. (2009). Ambulatory physical activity profiles of older adults. *Journal of Aging and Physical Activity*.

[16] Scott D., Blizzard L., Fell J., Jones G. (2009). Ambulatory activity, body composition, and lower-limb muscle strength in older adults. *Medicine and Science in Sports and Exercise*.

[17] Tudor-Locke C., Bassett D. R., Rutherford W. J. (2008). BMI-referenced cut points for pedometer-determined steps per day in adults. *Journal of Physical Activity and Health*.

[18] Centers for Disease Control and Prevention (2005). Trends in leisure-time physical inactivity by age, sex, and race/ethnicity—United States, 1994–2004. *Morbidity and Mortality Weekly Report*.

[19] Norman A., Bellocco R., Vaida F., Wolk A. (2003). Age and temporal trends of total physical activity in Swedish men. *Medicine and Science in Sports and Exercise*.

[20] Friedman H. S., Martin L. R., Tucker J. S., Criqui M. H., Kern M. L., Reynolds C. A. (2008). Stability of physical activity across the lifespan. *Journal of Health Psychology*.

[21] Colbert L. H., Matthews C. E., Havighurst T. C., Kim K., Schoeller D. A. (2011). Comparative validity of physical activity measures in older adults. *Medicine and Science in Sports and Exercise*.

